# PREDICTIVE EQUATIONS OF MAXIMUM OXYGEN CONSUMPTION BY SHUTTLE RUN TEST IN CHILDREN AND ADOLESCENTS: A SYSTEMATIC REVIEW

**DOI:** 10.1590/1984-0462/;2019;37;2;00016

**Published:** 2019-03-18

**Authors:** Francisco José de Menezes, Íncare Correa de Jesus, Neiva Leite

**Affiliations:** a Universidade Federal do Paraná, Curitiba, PR, Brazil.

**Keywords:** Cardiopulmonary Exercise Test, Cardiorespiratory fitness, Adolescents, Children, Teste de esforço, Aptidão cardiorrespiratória, Adolescentes, Crianças

## Abstract

**Objective:**

To systematically review the literature as for the level of evidence of predictive equations of VO_2peak_ through the 20-meter shuttle run test (20m-SRT) in children and adolescents.

**Data sources:**

Searches were conducted independently by two researchers, according to the procedures adopted by PRISMA, in the electronic databases MEDLINE via PubMed, ScienceDirect, Web of Science, LILACS and SciELO, for articles published until September 2017 in English and Portuguese. The inclusion criteria were: original studies, abstract available, using predictive equations of VO_2peak_ through 20m-SRT, conducted with adolescents and/or children, non-athletes, and mentioning correlation analysis between predicted and measured VO_2peak_. The level of evidence of equations was based on the risk of bias of the studies using the following criteria: sample number, sample characteristics, and statistical analysis.

**Data synthesis:**

Eighteen studies were selected, in which fifteen equations were found and analyzed. The studies had been conducted with samples composed of subjects of both sexes, aged 8 to 19 years. Equations of Léger and Matsuzaka had their level of evidence classified as high, and estimation ranged between r=0.54-0.90 and r=0.65-0.90. Equations by Ruiz, Barnett and Matsuzaka had their level of evidence classified as moderate, and estimation ranged between r=0.75-0.96, r=0.66-0.84 and r=0.66-0.89, respectively.

**Conclusions:**

Matsuzaka’s equation presented satisfactory parameters for estimates of VO_2peak_ in children and adolescents. Although not explored in equations, body adiposity and pubertal stage are significantly associated with cardiorespiratory fitness in children and adolescents.

## RESUMO

**Objetivo:**

Revisar sistematicamente na literatura o nível de evidência das equações preditivas do pico de consumo de oxigênio (VO_2pico_) por meio do teste de *shuttle run* de 20 metros (SR-20m) em crianças e adolescentes.

**Fonte de dados:**

As buscas foram conduzidas nas bases de dados eletrônicas Medical Literature Analysis and Retrieval System Online (MEDLINE) via PubMed, ScienceDirect, Web of Science, Literatura Latino-Americana e do Caribe em Ciências da Saúde (LILACS) e Scientific Electronic Library Online (SciELO), de agosto a setembro de 2017, nos idiomas inglês e português. Os critérios de inclusão utilizados foram: estudos originais, com resumo disponível, com equações para predição do VO_2pico_ por meio do SR-20m, adolescentes e/ou crianças, não atletas e com análise correlacional do VO_2pico_ predito e mensurado. O nível de evidência das equações foi caracterizado com base no risco de viés dos estudos, no qual se adotou os seguintes critérios: número da amostra, características da amostra e análise estatística.

**Síntese dos dados:**

Dezoito estudos foram selecionados, nos quais 12 equações foram encontradas e analisadas. Os estudos foram conduzidos com amostras de ambos os sexos com idades de oito a 19 anos. As equações de Léger e Matsuzaka foram classificadas com forte nível de evidência, com variação de amplitude de estimativa entre r=0,54-0,90 e r=0,65-0,90. Enquanto as equações Ruiz, Barnett e Matsuzaka foram consideradas de evidência moderada, com variação de amplitude de estimativa entre r=0,75-0,96, r=0,66-0,84 e r=0,66-0,89, respectivamente.

**Conclusões:**

A equação de Matsuzaka apresentou parâmetros satisfatórios para estimar o VO_2pico_ em crianças e adolescentes. Embora não explorados em equações, a adiposidade corporal e o estágio puberal demonstram associações relevantes com a aptidão cardiorrespiratória em crianças e adolescentes.

## INTRODUCTION

Cardiorespiratory fitness (CRF) is an important health marker in children and adolescents^1^, as it reflects cardiopulmonary efficiency for oxygen and musculoskeletal distribution during exercise or physical activity.^2^ ^,^ ^3^ Studies have shown that children with low CRF tend to maintain this condition over the years, which adversely affects their functional capacity to perform daily activities and quality of life.^4^ ^,^ ^5^ In addition, low CRF is associated with an increase in risk factors for cardiovascular diseases and metabolic changes related to pediatric morbidity and mortality in adults.^6^ ^,^ ^7^

Thus, CRF analysis is a measure of health status of the child and adolescent population.^6^ It provides relevant information to the diagnosis and prognosis of cardiometabolic risk factors.^6^ Moreover, it serves as an instrument in individualized therapeutics and exercise prescription.^8^ Oxygen consumption (VO_2_) is considered the main index to determine CRF.^9^ In children and adolescents, the peak of oxygen consumption (VO_2peak_) is generally used, defined as the peak of VO_2_ reached at the end of maximum effort period.^9^

VO_2peak_ can be measured by direct methods by ergospirometric analysis in maximum tests conducted in laboratory with different ergometers or in field, by sport activity simulation.^10^ ^,^ ^8^ From direct testings, authors have proposed equations that assess VO_2peak_ by indirect methods, which can be performed in maximum or submaximal tests, thus increasing practicality and reducing the costs of evaluations.^11^

In epidemiological studies, indirect field tests are mostly indicated because they usually require low cost, short time of execution and ease of simultaneous application in a larger number of individuals.^12^ ^,^ ^13^ The 20-meter shuttle run test (20m-SRT), conceived and described by Léger et al.^14^ for the adult population, is one of the field protocols most used in children and adolescents.^6^ 20m-SRT is considered a simple method, as it requires few equipment, can be performed in space-limited environments, and allows to assess several individuals at the same time, which can increase participants’ motivation.^2^ ^,^ ^6^ A systematic review including about 319,000 children and adolescents from 32 countries reported the performance achieved at the 20m-SRT as directly related to health indicators in children and adolescents.^5^

In the last decades, the 20m-SRT was included in several batches of physical fitness tests such as EUROFIT and FITNESSGRAM,^6^ resulting in the need to improve VO_2peak_ predictive equations through this test for the child and adolescent population.^15^ ^,^ ^16^ Equations were therefore developed using mathematical regression models or artificial neural networks, and including biological characteristics such as age, sex, body mass and performance in the test.^17^ ^,^ ^18^

On the other hand, prediction of VO_2peak_ by equations may vary in measurements depending on the characteristics of the sample, especially age group, stage of sexual maturation, gender, and body composition.^16^ ^,^ ^18^ So, in order for an equation to be considered appropriate, it must have adequate validity, that is, produce little variation range between estimate values.^19^ Batista et al.^19^ pointed out the relevance of analyzing the level of evidence of equations developed to estimate VO_2peak_ in children and adolescents, and contributed to this review in a more careful and orderly manner.

Therefore, it is not clear which equation establishes better accuracy for estimates based on the different characteristics of the child and adolescent population, or which variables are important to predict VO_2peak_, because so far, the findings of different studies have not been systematically analyzed. Thus, the objective of this study was to systematically review the literature to assess the level of evidence of equations intended to predict VO_2peak_ through the 20m-SRT in children and adolescents.

## METHOD

This work was conducted in compliance with recommendations by the Preferred Reporting Items for Systematic Review and Meta-analyzes: the PRISMA statement,^20^ from August to September 2017.

Five online databases were selected according to the field of knowledge and scientific relevance worldwide: Medical Literature Analysis and Retrieval System Online (MEDLINE) via PubMed, ScienceDirect, Web of Science, Latin American and Caribbean Literature in Health Sciences (LILACS), and Scientific Electronic Library Online (SciELO). We also searched the reference lists of articles selected that were related to the topic.

The search strategies were defined after identification and selection of search descriptors, based on DECS (BIREME health sciences descriptors) and MESH (Medical Subject Headings - controlled vocabulary used for indexing articles for PubMed). In this way, the following keywords were chosen in English and Portuguese: Cardiopulmonary Exercise Test, Oxygen consumption, Children and Adolescents. The keywords were combined using “AND” and/or “Boolean” terms and the period of study publication was set until September 2017.

After using the selected descriptors, the studies in duplicity were discarded and the inclusion (1, 2 and 3) and non-inclusion (4, 5 and 6) criteria were applied to screened studies, upon reading of the headings and abstracts:

- Original studies with transversal or longitudinal design, or clinical trial.
- Abstract accessible in the searched databases.
- Studies using equations for VO_2peak_ prediction for the 20m-SRT.
- Samples with adults and/or elderly people.
- Studies conducted with athletes.
- Absence of correlation analysis between VO_2peak_ predicted by indirect method and VO_2peak_ measured by direct method.

After this step, articles classified as eligible were read and analyzed in full; studies were excluded for several reasons: sample presenting a diagnosed pathology, sample made up of adults and adolescents who were analyzed together, adapted shuttle run protocol, no direct measurement of VO_2peak_, no correlation analysis, and/or no VO_2peak_ prediction.

The criteria for bias risk assessment were adapted by Batista et al.^19^ and three parameters were observed: number of participants, sample description and statistical analysis. In order to measure, each parameter was assigned a score of 0 to 2 points.

As for the number of participants, the studies were classified as “0”, when the sample had less than 10 participants; “1”, between 11 and 50 participants; or “2” more than 51 participants. Age, sex, health status, physical fitness level, pubertal status, body composition, physical activity level and ethnicity were considered when analyzing sample characteristics. Based on these aspects, the studies were scored as “0” when less than four characteristics were described; “1” for four characteristics; or “2” for more than four features. The studies were classified as “0” when presenting no regression analysis or error measures; “1”, when presenting regression analysis and/or error measures; and “2” when more than three statistical analyzes were present, or Bland-Altman plot and/or analysis of variance (ANOVA) of repeated measurements. The studies were all categorized according to the scores received: high risk of bias (0-2 points), moderate risk of bias (3 and 4 points) and low risk of bias (5 and 6 points).

Subsequently, the validity of the identified equations was assessed based on the evidence-level criteria expressed by Castro-Piñero et al.:^21^

- Strong evidence: equations validated by three or more studies with low risk of bias.
- Moderate evidence: equations validated by two studies with low risk of bias.
- Limited evidence: equations validated by studies with high risk of bias, inconsistent results among several studies, regardless of risk of bias, or the results of a single study.

Some characteristics of the samples were highlighted in the studies, such as age, gender and number of subjects. The values of correlation coefficient (r) and standard estimation error (SEE) in mL/kg.min were extracted when available. Estimate range variation (ΔER) of each equation was determined by the description from the lowest to the highest correlation coefficient obtained by the equation between the studies. To facilitate identification, we chose to name the equations with the name of the first author of the study in which it was validated. When one author had identified two or more equations in a single study, each equation was accompanied by (a), (b) or (c).

The steps of the process of research, selection, analysis, application of bias risk parameters, and data extraction were independently performed by two researchers (FJMJ and ICJ), and, in case of disagreement, a third researcher (NL) was asked to decide on divergent points.

## RESULTS

**Figure 1:**
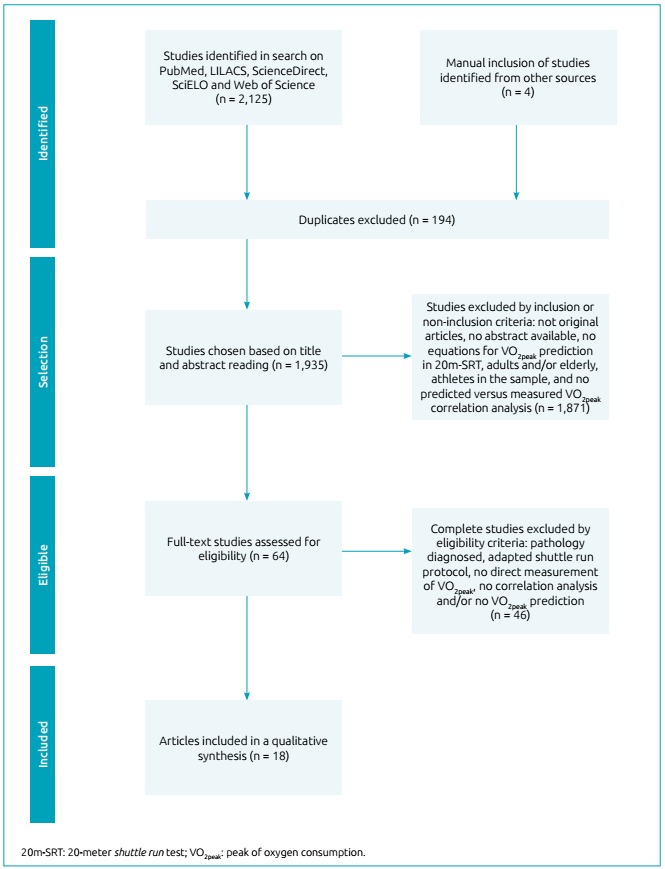
PRISMA flowchart.

In total, 2,125 studies were found using the combination of selected descriptors, but 194 were discarded for being duplicates. Afterwards, the inclusion and non-inclusion criteria were applied and 64 studies were considered eligible in full, ending the selection with 14 articles for qualitative synthesis. In addition, four studies relating to the theme identified in other reference lists of articles selected were included, so 18 studies were selected. The process of studies selection is outlined in Figure 1.

Following criteria adapted by Batista et al.,^19^ nine studies were classified as low risk of bias,^17^ ^,^ ^21^ ^,^ ^22^ ^,^ ^23^ ^,^ ^24^ ^,^ ^25^ ^,^ ^26^ ^,^ ^27^ ^,^ ^36^ and other nine as moderate risk of bias.^15^ ^,^ ^16^ ^,^ ^18^ ^,^ ^29^ ^,^ ^30^ ^,^ ^31^ ^,^ ^32^ ^,^ ^33^ ^,^ ^34^ ^,^ ^35^ The details of risk assessment criteria are shown in Table 1.

**Table 1:** Classification of bias risk of each study.

Studies	Number of subjects	Sample characteristics	Statistical analysis	Bias risk
Léger et al.^29^	2	0	1	Moderate
Liu et al.^30^	1	2	1	Moderate
Barnett et al.^31^	2	1	1	Moderate
Pitetti et al.^32^	2	1	1	Moderate
Suminski et al.^22^	2	2	1	Low
Matsuzaka et al.^23^	2	2	2	Low
Mahar et al.^33^	2	1	1	Moderate
Ruiz et al.^17^	2	1	2	Low
Ruiz et al.^15^	1	1	2	Moderate
Boiarskaia et al.^34^	2	1	0	Moderate
Mahar et al.^18^	2	1	1	Moderate
Melo et al.^24^	2	2	1	Low
Silva et al.^25^	2	2	2	Low
Batista et al.^26^	2	2	2	Low
Quinart et al.^37^	1	2	2	Low
Burns et al.^27^	2	1	2	Low
Ernesto et al.^28^	2	1	2	Low
Sain-Maurice et al.^16^	2	1	1	Moderate

5 or 6: low bias risk; 3 or 4: moderate risk of bias; 1 or 2: high risk of bias.

The samples of studies selected had subjects aging 8 to 19 years, most of them with nutritional status classified as eutrophic, except for two overweight studies.^22^ ^,^ ^36^

We identified studies that aimed to develop equations using variables in mathematical regression models,^18^ ^,^ ^23^ ^,^ ^25^ ^,^ ^27^ ^,^ ^29^ ^,^ ^31^ ^,^ ^33^ ^,^ ^35^ ^,^ ^36^ and artificial neural networks.^17^ ^,^ ^25^ The variables used in equations were gender,^17^ ^,^ ^18^ ^,^ ^23^ ^,^ ^25^ ^,^ ^31^ ^,^ ^33^ ^,^ ^35^ ^,^ ^36^ age, ^17^ ^,^ ^23^ ^,^ ^25^ ^,^ ^27^ ^,^ ^29^ ^,^ ^31^ ^,^ ^33^ ^,^ ^36^ body mass index (BMI),^18^ ^,^ ^23^ ^,^ ^25^ ^,^ ^33^ ^,^ ^35^ ^,^ ^37^ body mass,^17^ ^,^ ^25^ ^,^ ^31^ ^,^ ^33^ stature^17^ ^,^ ^25^ and triceps skinfold^31^, besides performance in 20m-SRT (final speed in km/h,^23^ ^,^ ^29^ ^,^ ^31^ ^,^ ^36^ number of laps,^18^ ^,^ ^23^ ^,^ ^28^ ^,^ ^33^ ^,^ ^35^ number of stages^17^ ^,^ ^25^ and number of laps squared.^18^ The equations identified had their characteristics detailed in Tables 2 and 3.

**Table 2:** Equations for prediction of maximal oxygen consumption upon the 20-meter shuttle run test in children and adolescents (equations by Léger et al., Ruiz et al., Barnett et al.)

Equation	Study	(n) age range	r (SEE mL/kg.min) (predicted versus measured VO_2_)			Variables in equation
			♀ ♂	♂	♀	
Léger et al.^29^	Léger et al.^29^	(188) 8-19	0.71 (5.9)			Age; final speed
	Liu et al. ^30^	(62) 12-15	0.72 (5.2)			
	Barnett et al.^31^	(55) 12-17	0.72 (5.4)			
	Pitetti et al.^32^	(61) 8-15	0.57			
	Suminski et al.^22^	(125) 10-12	0.62 (3.9)	0.58 (4.7)	0.55 (3.1)	
	Suminski et al.^22^	(81) 10-12	0.54 (4.2)			
	Suminski et al.^22^*	(44) 10-12	0.81 (3.2)			
	Mahar et al.^33^	(135) 12-14	0.54 (6.6)			
	Ruiz et al.^17^	(193) 13-19	0.90 (4.2)			
	Ruiz et al.^15^	(48) 13-19	0.58 (6.5)			
	Boiarskaia et al.^34^	(135) 12-14	0.54	0.46	0.39	
	Mahar et al.^18^	(244) 10-16	0.58 (7.6)			
	Melo et al.^24^	(90) 8-10	0.88			
	Silva et al.^25^	(114) 10-18	0.67 (7.1)			
	Batista et al.^26^	(115) 11-13	0.60 (7.5)	0.60 (7.7)	0.49 (6.4)	
	Ernesto et al.^28^	(90) 13-17		0.76 (4.1)	0.53 (2.4)	
Ruiz et al.^17^	Ruiz et al.^17^	(193) 13-19	0.96 (2.8)			Gender; age; BM; stature; stage
	Ruiz et al.^15^	(48) 13-19	0.75 (5.3)			
	Silva et al.^25^	(114) 10-18	0.86 (6.2)			
Barnett et al.^31^ (a)	Barnett et al.^31^	(55) 12-17	0.84 (3.7)			Gender; BM; final speed
	Ruiz et al.^15^	(48) 13-19	0.75 (5.3)			
	Mahar et al.^18^	(244) 10-16	0.66 (7.0)			
	Melo et al., ^24^	(90) 8-10	0.68			
	Batista et al.^26^	(115) 11-13	0.79 (5.81)	0.77 (6.0)	0.72 (5.1)	
Barnett et al.^31^ (b)	Barnett et al.^31^	(55) 12-17	0.82 (4.0)			Gender; age; final speed
	Ruiz et al.^15^	(48) 13-19	0.72 (5.6)			
	Mahar et al.^18^	(244) 10-16	0.64 (7.2)			
	Silva et al.^25^	(114) 10-18	0.71 (6.8)			
	Ernesto et al.^28^	(90) 13-17		0.76 (4.1)	0.66 (4.2)	
Barnett et al.^31^ (c)	Barnett et al.^31^	(55) 12-17	0.85 (3.7)			Gender; triceps skinfold; final speed
	Melo et al.^24^	(90) 8-10	0.62			

n: sample size; r: correlation coefficient; SEE: standard estimate error; VO_2_: oxygen consumption; ♀: male; ♂: female; *overweight; BM: body mass;

**Table 3:** Equations for prediction of maximal oxygen consumption upon the 20m-SRT test in children and adolescents (Matsuzaka et al., Mahar et al., Silva et al., Burns et al., Fernhall et al., And Quinart et al.)

Equation	Study	(n) age range	r (SEE mL/kg.min) (predicted versus measured VO_2_)			Variables in equation
			♀ ♂	♂	♀	
Matsuzaka et al.^23^ (a)	Matsuzaka et al.^23^	(132) 8-17	0.90 (3.3)			Gender; age; BMI; final speed
	Ruiz et al.^15^	(48) 13-19	0.73 (5.5)			
	Mahar et al.^18^	(244) 10-16	0.65 (7.1)			
	Melo et al.^24^	(90) 8-10	0.72			
	Batista et al.^26^	(115) 11-13	0.77 (5.9)	0.80 (5.8)	0.69 (5.3)	
Matsuzaka et al.^23^ (b)	Matsuzaka et al.^23^	(132) 8-17	0.89 (3.4)			Gender; age; BMI; laps
	Mahar et al.^18^	(244) 10-16	0.66 (7.0)			
	Melo et al.^24^	(90) 8-10	0.80			
Mahar et al.^33^ (a)	Mahar et al.^33^	(135) 12-14	0.64 (6.44)			Gender; laps; BM
	Boiarskaia et al.^34^	(135) 13	0.57	0.45	0.39	
	Mahar et al.^18^	(244) 10-16	0.66 (6.99)			
	Batista et al.^26^	(115) 11-13	0.80 (5.69)	0.77 (6.11)	0.71 (5.2)	
Mahar et al.^33^ (b)	Mahar et al.^33^	(135) 12-14	0.64 (6.4)			Gender; age; BMI; laps
	Boiarskaia et al.^34^	(135) 13	0.65	0.56	0.51	
	Mahar et al.^18^	(244) 10-16	0.71 (6.6)			
	Burns et al.^27^	(90) 13-16	0.78			
Mahar et al.^18^ (quadrática)	Boiarskaia et al.^34^	(135) 13	0.67	0.62	0.52	Gender; BMI; laps; laps²
	Mahar et al.^18^	(244) 10-16	0.73 (6.3)			
	Burns et al.^27^	(90) 13-16	0.74			
Silva et al.^25^ (a)	Silva et al.^25^	(114) 10-18	0.80 (5.7)			Gender; BMI; Stage
Silva et al.^25^ (b)	Silva et al.^25^	(114) 10-18	0.86 (5.0)			Gender; age; BM; stature; BMI; stage
Burns et al.^27^	Burns et al.^27^	(90) 13-16	0.77			Age; laps
	Saint-Maurice et al.^16^	(310) 10-18		0.36	0.42	
Fernhall et al.^35^	Pitetti et al.^32^	(51) 8-15	0.66			Gender; BMI; laps
	Melo et al.^24^	(90) 8-10	0.56			
Quinart et al.^36^	Quinart et al.^37^*	(30) 12-17	0.77			Gender; age; BMI; final speed

n: sample size; r: correlation coefficient; SEE: standard estimate error; VO_2_: oxygen consumption; ♀: male; ♂: female; *overweight; BMI: body mass index; laps: number of laps; laps²: number of laps squared; MC: body mass.

In addition, some studies had a cross-validation of equations as objective: Léger,^15^ ^,^ ^17^ ^,^ ^18^ ^,^ ^22^ ^,^ ^24^ ^,^ ^25^ ^,^ ^26^ ^,^ ^28^ ^,^ ^30^ ^,^ ^31^ ^,^ ^32^ ^,^ ^33^ ^,^ ^34^ Barnett (a),^15^ ^,^ ^18^ ^,^ ^24^ ^,^ ^26^ Barnett (b),^15^ ^,^ ^18^ ^,^ ^25^ ^,^ ^28^ Barnett (c),^24^ Matsuzaka (a),^15^ ^,^ ^18^ ^,^ ^24^ ^,^ ^26^ Matsuzaka (b),^18^ ^,^ ^24^ Mahar (a),^18^ ^,^ ^26^ ^,^ ^34^ Mahar (b),^18^ ^,^ ^27^ ^,^ ^34^ Mahar (squared),^27^ ^,^ ^34^ Burns^16^ and Fernhall.^23^ ^,^ ^32^ Among all equations, two had a strong level of evidence,^23^ ^,^ ^29^ three had moderate level^17^ ^,^ ^23^ ^,^ ^31^ and seven had limited level of evidence.^18^ ^,^ ^27^ ^,^ ^31^ ^,^ ^33^ ^,^ ^35^

Among the equations with strong level of evidence, Léger^29^ was the most commonly applied in cross-validations, however it shows considerable ΔER and lower values of estimates for girls. The equation by Matsuzaka (a)^23^ is considered strong-evidence, and able to generate estimates with lower ΔER and higher correlation values for boys.

As for moderate evidence, the equation by Ruiz^16^ showed a low ΔER, while Barnett’s (a)^31^ and Matsuzaka’s (b)^23^ resulted in high association values, but low ΔER, respectively. In addition, Barnett’s equation (a)^31^ had higher correlation values for girls.

Finally, the equations by Barnett (b),^31^ Barnett (c),^31^ Mahar (a),^33^ Mahar (b),^33^ Mahar (squared),^18^ Silva (a),^25^ Silva (b),^25^ Burns,^27^ Fernhall^35^ and Quinart^36^ were classified as limited evidence. The level of evidence of equations and respective ΔER are listed in Table 4.

**Table 4:** Classification of level of evidence and estimate range variation of the equations

Equations	Low risk of bias	Moderate risk of bias	Level of evidence	∆ER All studies	∆ER Studies with low risk of bias
Léger et al.^29^	5	9	Strong	r=0.54-0.90	r=0.54-0.90
Ruiz et al.^17^	2	1	Moderate	r=0.75-0.96	r=0.86-0.96
Barnett et al.^31^ (a)	2	3	Moderate	r=0.66-0.84	r=0.68-0.79
Barnett et al.^31^ (b)	2	3	Limited	r=0.64-0.82	r=0.71
Barnett et al.^31^ (c)	1	1	Limited	r=0.62-0.85	r=0.62
Matsuzaka et al.^23^ (a)	3	2	Strong	r=0.65-0.90	r=0.72-0.90
Matsuzaka et al.^23^(b)	2	1	Moderate	r=0.66-0.89	r=0.80-0.89
Mahar et al.^33^ (a)	1	3	Limited	r=0.57-0.80	r=0.80
Mahar et al.^33^ (b)	1	3	Limited	r=0.64-0.78	r=0.78
Mahar et al.^18^ (quadrática)	1	2	Limited	r=0.67-0.74	r=0.74
Silva et al.^25^ (a)	1	0	Limited	r=0.80	r=0.80
Silva et al.^25^ (b)	1	0	Limited	r=0.86	r=0.86
Burns et al.^27^	1	1	Limited	r=0.77	r=0.77
Fernhall et al.^35^	1	1	Limited	r=0.56-0.66	r=0.56
Quinart et al.^36^	1	0	Limited	r=0.77	r=0.77

∆ER: estimate range variation; strong evidence: more than three studies with low risk of bias; moderate evidence: two studies with low risk of bias; limited evidence: several studies with high risk of bias, wide range of variation or only one study.

## DISCUSSION

This systematic review gathered 18 studies in which fifteen equations were identified. Among these, different variables were employed, including sample characteristics and performance in 20m-SRT. Two equations had a strong level of evidence,^23^ ^,^ ^29^ three were classified as moderate evidence^17^ ^,^ ^23^ ^,^ ^31^, and nine as limited evidence.^18^ ^,^ ^25^ ^,^ ^27^ ^,^ ^31^ ^,^ ^33^ ^,^ ^35^ Our findings show that Matsuzaka’s (a)^23^ equation tends to have higher predictive reliability and a high level of evidence for both genders and may be a potential equation to estimate the VO_2peak_ in eutrophic boys.

As previously presented, children and adolescents with high VO_2peak_ levels tend to have risk factors related to cardiovascular diseases, obesity and the metabolic syndrome reduced.^5^ ^,^ ^7^ Thus, the accuracy of equations to estimate VO_2peak_ is relevant, since it provides valuable information for the diagnosis and prognosis of cardiometabolic risk factors.^6^ ^,^ ^8^ Access to a practical and inexpensive method is important; the 20m-SRT has fulfilled this requirement with strong level of evidence.^6^ ^,^ ^19^ This test requires cheap resources and infrastructure that is easily accessible in schools, clubs and academies. In addition, it can be considered practical and efficient, as it allows the evaluation of several people at the same time.^6^

According to our findings, Léger’s equation^29^ was primary to estimate VO_2peak_ in children and adolescents in the literature. This equation, which uses age and performance in 20m-SRT as variables, was more popular in studies and presented strong evidence. However, it presents a considerable ΔER between correlation values, being frequently inferior to r = 0.60.^15^ ^,^ ^18^ ^,^ ^22^ ^,^ ^32^ ^,^ ^33^ ^,^ ^34^ This variation can be explained by differences in gender between subjects in the sample. Although Léger et al.^29^ found no significant predictive value for gender, other studies demonstrate a strong association between this component and cardiorespiratory fitness in children and adolescents.^18^ ^,^ ^32^

On the other hand, the Matsuzaka’s equation (a)^23^, with strong evidence, obtained values of estimate validity with lower ΔER. The authors^23^ included gender, age, BMI and 20m-SRT performance in the equation, that is, theirs was the first study to include BMI in prediction equations. This equation can be considered the one with greater estimation precision.

On the other hand, although the equations by Ruiz,^17^ Barnett (a)^31^ and Matsuzaka (b)^23^ were classified with moderate level of evidence, they showed relevant estimates of validity. In particular, Ruiz’s^17^ equation presented the lowest ΔER among the estimation results. In addition, it matched Matsuzaka’s (a)^23^, taking the greater number of characteristics of the sample included into account (sex, age, body mass, height and 20m-SRT performance). This equation was evaluated by a few studies, but seems to be a promising tool that should be better studied.

When considering only studies with low risk of bias, findings become more evident. Léger’s equation^29^ continues to present higher ΔER compared to Matsuzaka’s (a)^23^, among equations with strong evidence; Ruiz^17^ obtained higher correlation values and lower ΔER, in comparison to the other equations of moderate evidence.

When analyzing data by gender in samples, only the Léger’s^29^ equation reached strong evidence, despite having low correlation values and high ΔER, showing underestimation of VO_2peak_ prediction for females and males. Therefore, it was not possible to define the validity of the specific equations by gender, since few studies have provided isolated correlational information and analysis with this variable. Despite this, Barnett’s (a)^31^ may be a potential equation to estimate VO_2peak_ in girls and Matsuzaka’s (a)^23^ in boys, since they were shown to have higher correlational values for the respective groups.

Among equations with strong and moderate level of evidence, the Matsuzaka’s (a),^23^ Matsuzaka’s (b)^23^ and Ruiz’s^17^ equations were the ones that used the largest number of variables from the sample and obtained a lower ΔER with high correlation values. Léger^29^ and Barnett (a)^31^ inserted fewer variables and found higher ΔER values. The use of more than one characteristic of the sample, such as gender, body mass, stature or BMI, in equations tends to result in higher values of association between predicted and measured VO_2peak_. This trend was also noted in other studies.^37^ ^,^ ^38^ From this point of view, moderate associations between VO_2peak_ and BMI, body mass and gender were identified.^25^ ^,^ ^32^ ^,^ ^33^

According to Saint-Maurice et al.,^38^ BMI tends to have a larger influence on CRF in children and adolescents, which can explain 30 to 34% of the variance between VO_2peak_ estimates found with predictive equations. In this perspective, equations that do not take BMI into account tend to overestimate the CRF of individuals in high nutritional status.^38^

Although not yet explored in prediction equations, the body fat percentage shows a significant association with CRF in both children and adolescents.^39^ Correlational values of r=-0.60 for both genders, r=-0.48 to -0,53 for boys and r=-0.24 to -0.40 for girls evidence this variable as a strong predictor for males and moderate predictor for females.^27^ ^,^ ^40^ ^,^ ^41^

Although chronological age has often been used to characterize physical fitness profile, the different stages of sexual maturation tend to relate to different physical fitness characteristics in children and adolescents.^42^ ^,^ ^43^ Girls, specifically, demonstrate significant differences in CRF in different stages of sexual maturation, often presenting decreased VO_2peak_ as their stages of sexual maturation progress.^44^ However, this variable has not yet been tested in prediction equations.

In addition, children and adolescents of different economic classes, sedentary behavior profiles and habitual physical activity levels may present differences as to health-related parameters.^40^ ^,^ ^44^ ^,^ ^45^ However, information about the use of these variables to predict VO_2peak_ in children and adolescents is still limited, and new studies on the topic should be developed to better understand the influence of these variables on VO_2peak_ prediction.

This study has some limitations for analysis that should be listed, such as lack of information on sample characteristics, adiposity level, level of physical activity and sedentary behavior; especially related to correlation analyses for samples adjusted by gender. These limitations have turned the identification of the best predictive equation for different groups of children and adolescents into a challenge.

Therefore, future research should be able to provide more information on the sample, such as ethnicity, length of time with sedentary behavior, physical activity level, aspects of body composition and stage of sexual maturation, as well as to promote correlations with CRF. Thus, doubts about the association between these variables and VO_2peak_ prediction can be better understood, allowing more accurate equations to be elaborated. It is also important that further research be conducted to verify the reproducibility of the equations proposed by Ruiz,^17^ Barnett (a),^31^ Matsuzaka (a)^23^ and Matsuzaka (b),^23^ identified in this review as promising but poorly explored in studies. These should be tested for both children and adolescents with different nutritional status, as well as gender-specific variations.

In the present study, we were able to point out the equations with better validity of VO_2peak_ prediction for children and adolescents, as well as to identify aspects that hindered a more satisfactory analysis to elect a definitive equation. The suggestions presented in this review contribute to a more accurate elaboration and description of future studies, which contributes to the expansion of the scientific and practical knowledge about predictive equations for VO_2peak_ in children and adolescents.

In conclusion, our findings suggest that using more than one sample feature in equations tends to exert higher association values between predicted and measured VO_2peak_. Matsuzaka’s equation (a), in this sense, tends to have the strongest level of evidence with greater precision of estimation in children and adolescents. Although not explored in prediction equations, body fat percentage and sexual maturation stage are shown to have relevant associations with CRF in children and adolescents and further analyses of these variables in other equations are encouraged. However, new research should be conducted to evaluate the reproducibility of equations considered by this review as promising, as well as to improve the understanding about the relationship between anthropometric variables, body composition components and sexual maturation stages with VO_2peak_ prediction in children and adolescents.
